# Recent Advances and Ongoing Challenges in the Diagnosis of Microbial Infections by MALDI-TOF Mass Spectrometry

**DOI:** 10.3389/fmicb.2018.01097

**Published:** 2018-05-29

**Authors:** Walter Florio, Arianna Tavanti, Simona Barnini, Emilia Ghelardi, Antonella Lupetti

**Affiliations:** ^1^Dipartimento di Ricerca Traslazionale e delle Nuove Tecnologie in Medicina e Chirurgia, Università di Pisa, Pisa, Italy; ^2^Dipartimento di Biologia, Università di Pisa, Pisa, Italy; ^3^Azienda Ospedaliero-Universitaria Pisana, Pisa, Italy

**Keywords:** blood culture, MALDI-TOF mass spectrometry, bacterial identification, fungal identification, antimicrobial resistance

## Abstract

Timeliness and accuracy in the diagnosis of microbial infections are associated with decreased mortality and reduced length of hospitalization, especially for severe, life-threatening infections. A rapid diagnosis also allows for early streamlining of empirical antimicrobial therapies, thus contributing to limit the emergence and spread of antimicrobial resistance. The introduction of matrix-assisted laser desorption/ionization-time of flight (MALDI-TOF) mass spectrometry (MS) for routine identification of microbial pathogens has profoundly influenced microbiological diagnostics, and is progressively replacing biochemical identification methods. Compared to currently used identification methods, MALDI-TOF MS has the advantage of identifying bacteria and yeasts directly from colonies grown on culture plates for primary isolation in a few minutes and with considerable material and labor savings. The reliability and accuracy of MALDI-TOF MS in identification of clinically relevant bacteria and yeasts has been demonstrated by several studies showing that the performance of MALDI-TOF MS is comparable or superior to phenotypic methods currently in use in clinical microbiology laboratories, and can be further improved by database updates and analysis software upgrades. Besides microbial identification from isolated colonies, new perspectives are being explored for MALDI-TOF MS, such as identification of pathogens directly from positive blood cultures, sub-species typing, and detection of drug resistance determinants. In this review, we summarize the state of the art in routine identification of microbial pathogens by MALDI-TOF MS, and highlight recent advancements of this technology in special applications, such as strain typing, assessment of drug susceptibility, and detection of virulence factors.

## Introduction

The application of mass spectrometry (MS) to bacterial identification was firstly proposed by Anhalt and Fenselau (1975), based on the observation that different mass spectra were obtained from bacterial extracts of different species. However, the hard ionization allowed detecting only bacterial lipids, which limited the possibility to discriminate bacteria at species level. The analysis of high molecular mass biomolecules, such as polypeptides, became possible only in the second half of the 1980s, after the development of soft ionization techniques (Tanaka et al., 1988). Holland et al. (1996) showed that specific spectral fingerprints could be obtained from whole bacterial cells by matrix-assisted laser desorption/ionization time-of-flight (MALDI-TOF) MS analysis, paving the way for microbial identification by MS. Subsequent developments have mainly focused on software and database improvements, making MALDI-TOF MS an increasingly robust and accurate tool for microbial identification (Kliem and Sauer, 2012).

Matrix-assisted laser desorption/ionization is a soft ionization technique allowing ionization and vaporization of intact, high molecular mass biomolecules. The analyzed spectrum is usually between 2 and 20 kDa, a molecular mass range dominated by ribosomal proteins (Ryzhov and Fenselau, 2001). The suppression of ionization of high-mass/acidic/low abundant proteins in presence of low-mass/basic/abundant ones yields MALDI mass spectra containing signals in the 2–20 kDa range. Microbial identification is achieved by comparing the observed spectrum with those stored in a database. Using a MALDI Biotyper-based identification (Bruker Daltonics), a score value between 0 and 3 is calculated by the software on the basis of peak similarities between the observed spectrum and stored reference spectra; score values of ≥2.00 indicate high confidence species ID, score values ≥1.70 and <2.00 are reported as low confidence ID, and scores <1.7 are considered as unreliable (no ID). However, the results of several studies suggest that lower score cut-off values might be applied for identification of many bacterial species without compromising accuracy (Richter et al., 2012; Szabados et al., 2012; Barnini et al., 2015), and it has been suggested that species-specific score cut-off values might be defined and applied in a species-specific manner (Richter et al., 2012) in order to optimize MALDI-TOF MS identification.

With the Vitek MS system (bioMérieux, Marcy-l’Étoile, France), the acquired mass spectrum is divided into 1,300 bins, and a score is generated and converted to a probability percentage and confidence value by comparing the peak patterns with those in the database; threshold values for reliable species identification are fixed in the binning algorithm^1^.

## Identification of Bacteria

Conventional microbiological methods for bacterial identification involve culture, microscopic examination and biochemical testing. These procedures can be accurate and reliable, at least for most of classical microbial pathogens, but they are also time consuming, laborious, and require specifically trained personnel for correct interpretation of results. Conversely, MS is rapid, reliable and easy to perform, it is reproducible (Westblade et al., 2015), and has the potential to replace or complement conventional biochemical methods for identification of bacteria, especially for emerging opportunistic pathogens that may be difficult to identify on the sole basis of biochemical tests. A small amount of bacterial biomass is required, and sample preparation for analysis is, in most cases, rapid and easy to perform. The spectral fingerprint obtained is compared with a database of reference spectra and a result is generated on the basis of peak similarities (Böhme et al., 2012).

Matrix-assisted laser desorption/ionization-time of flight MS has been shown able to achieve correct identification rates comparable to or higher than those of the BD Phoenix (Becton Dickinson) and Vitek 2 (bioMérieux) automated ID systems, besides shortening identification time from 24 to 48 h to just a few minutes (Dupont et al., 2010; Guo et al., 2014). The Bruker Biotyper (Bruker Daltonics) and Vitek MS (bioMérieux) are two of the most widely used MALDI-TOF MS systems for bacterial identification in diagnostic microbiology laboratories. In the overall, the two systems have shown similar performances (Cherkaoui et al., 2010; Croxatto et al., 2012; Marko et al., 2012; Martiny et al., 2012; Alby et al., 2013; Karpanoja et al., 2014; Mather et al., 2014; Lévesque et al., 2015). In a first evaluation of the accuracy of the Bruker system for routine identification of bacteria, the system correctly identified 95.4% of the 1660 bacterial isolates tested, 84.1% at species level and 11.3% at genus level (Seng et al., 2009). Lack of identification and misidentification were mainly due to insufficient database entries for some bacterial species but these limitations could be overcome by the addition of spectra of successfully identified clinical isolates into commercial databases (Seng et al., 2009; Fernández-Olmos et al., 2012; Nomura, 2015; Carrasco et al., 2016; Marín et al., 2017).

In the overall, the quality of the reference database is a fundamental prerequisite for correct identification of clinical isolates, and highly reliable ID results can be achieved by database incorporation of spectra of ascertained microbial species, including rare bacterial species (Seng et al., 2013). Improvement of database entries with multiple spectra of well-characterized species has been shown to yield high identification rates for *Clostridium* spp., *Mycobacterium* spp. (Pignone et al., 2006; El Khechine et al., 2011) and *Helicobacter pylori* (Wieser et al., 2012), amongst others. However, for microorganisms with complex cell wall composition such as *Mycobacterium* spp., the accuracy of identification is often sub-optimal (Cao et al., 2018), and specific pretreatment may be required for protein extraction before MALDI-TOF MS analysis can be effectively performed (Park et al., 2016; Moreno et al., 2018). Moreover, identification of some bacterial species may be still problematic, especially for Gram-positive bacteria such as *Streptococcus pneumoniae* and members of the *Streptococcus oralis/mitis* group (Marín et al., 2017; Slotved et al., 2017), and *Listeria* spp. (Farfour et al., 2012) but also for some Gram-negative bacteria such as *Shigella* spp., having low rate of differences in their ribosomal protein sequence with *Escherichia coli* (Khot and Fisher, 2013), some *Enterobacter* spp. (Elbehiry et al., 2017), and some strains of *Stenotrophomonas maltophilia* (Homem de Mello de Souza et al., 2014); in addition, MALDI-TOF MS identification of anaerobes at species level may be problematic (Rodríguez-Sánchez et al., 2016).

Microbial identification in polymicrobial samples by MALDI-TOF MS is also challenging, though some studies have shown that can be feasible (Mahe et al., 2014; Sakarikou et al., 2018). In many cases, the prevailing microorganism among those grown in blood culture (BC) can be correctly identified (Gray et al., 2013).

On the other hand, in a few cases, MALDI-TOF MS can provide an excellent means to identify bacterial species that biochemically-based microbiology has difficulty to distinguish, as in the case of *Haemophilus influenzae* and *Haemophilus haemolyticus* (Zhu et al., 2013).

Matrix-assisted laser desorption/ionization MS has also been evaluated for direct identification of microorganisms in complex biological fluids such as urine and BCs. Regarding urine, implementation of MALDI-TOF MS together with total laboratory automation significantly reduced turnaround time to identification, AST report, and preliminary negative results (Theparee et al., 2018). Microbial identification directly from positive BCs is described below.

Of interest, a comprehensive analysis of the *Treponema pallidum* proteome has been recently carried out (Osbak et al., 2016), laying the foundations for a possible, future MALDI-TOF MS-based identification of this important bacterial pathogen.

## Rapid Identification of Bacteria From Positive Blood Cultures

The short time and tiny amount of microbial biomass required for analysis make MALDI-TOF MS one of the most promising techniques for the identification of microbial pathogens directly from positive BCs (La Scola and Raoult, 2009; Moussaoui et al., 2010; Lagacé-Wiens et al., 2012; Clerc et al., 2013; Jamal et al., 2013; Machen et al., 2014; Barnini et al., 2015). To this purpose, separation of microorganisms from blood cells is a critical step for successful identification of bacteria in monomicrobial BCs by MALDI-TOF MS (Christner et al., 2010). Several sample preparation protocols have been proposed to separate bacteria from host cells and proteins through cell lysis and/or differential centrifugation steps (Christner et al., 2010; Stevenson et al., 2010; Schubert et al., 2011), and differences in sample processing methods may account, at least in part, for the observed variability in correct identification rates (Loonen et al., 2012). An alternative to cell lysis and/or differential centrifugations is purification of bacteria from BC using serum separator tubes (SSTs), which is simple, rapid and yields a relatively high recovery of bacteria (Lupetti et al., 2010; Barnini et al., 2015).

It should be mentioned that, due to the additional processing time, most microbiological laboratories use these methods in batches, thus reducing the time-saving achievable by a faster method. A simple, rapid and reliable method that does not necessarily require to work in batches has been proposed by Barnini et al. (2015).

Several studies indicate that integrating rapid MALDI-TOF MS identification with antimicrobial stewardship intervention for streamlining empirical antimicrobial therapy in patients with bloodstream infections can improve patient outcomes significantly, compared to reporting ID results alone (Huang et al., 2013; Nagel et al., 2014; Perez et al., 2014; Lockwood et al., 2016). In addition, rapid MALDI-TOF MS identification results can be used optimally in combination with rapid molecular and/or phenotypic methods for the detection of antimicrobial resistance (Lupetti et al., 2013; Machen et al., 2014; Barnini et al., 2016).

Adopting lower score cut-offs than those indicated by the systems’ manufacturers, preferably complemented by additional validation criteria, has been often proposed for microorganisms recovered from positive BCs (Idelevich et al., 2014; Barnini et al., 2015), due to the high background in these samples.

## Assessment of Antimicrobial Resistance and Strain Typing

Matrix-assisted laser desorption/ionization MS can be useful for the detection of bacterial resistance to β-lactam antibiotics mediated by β-lactamases (Burckhardt and Zimmermann, 2011; Hrabák et al., 2011, 2012; Hooff et al., 2012; Sparbier et al., 2012). The hydrolysis of the β-lactam ring can be detected by MS as the disappearance of the original mass peak of the antibiotic and appearance of the corresponding hydrolytic products (Jung et al., 2014).

Several studies have been undertaken aimed at identifying proteomic differences between methicillin-resistant and methicillin-sensitive *Staphylococcus aureus* and/or between vancomycin-resistant and vancomycin-sensitive *Enterococcus faecium*, and discriminating methicillin-resistant *S. aureus* lineages using MALDI-TOF MS (Du et al., 2002; Schlebusch et al., 2010; Gagnaire et al., 2012; Josten et al., 2013; Sparbier et al., 2013; Wang et al., 2013). However, a study aimed at identifying lineage-specific biomarker peaks revealed only limited possibilities to differentiate *S. aureus* as well as *E. faecium* below the species level (Lasch et al., 2014), suggesting that phenotypic differences may not always be detectable in a MALDI TOF mass spectrum.

In an attempt to overcome these difficulties, an indirect method has been established to detect methicillin resistance in a particular subset of *S. aureus* isolates. This method is based on the evidence that some MRSA strains produce a phenol-soluble protein toxin, called PSM-mec, belonging to the modulin family (Chatterjee et al., 2011). The presence of PSM-mec could be detected by MALDI-TOF MS as a 2415 ± 2 m/z peak (Rhoads et al., 2016).

The *psm-mec* gene, coding for the PSM-mec peptide, is present in the class A mec gene complex, which is carried by staphylococcal cassette chromosome mec (SCCmec) types II, III, and VIII (Chatterjee et al., 2011), and may be present in up to half of MRSA isolates (David et al., 2014; Rhoads et al., 2016). PSM-mec does not cause methicillin resistance, and its biological function is still under investigation. Absence of this toxin does not imply methicillin susceptibility and, therefore, additional methods are still required to confirm MSSA isolates. However, expression of PMS-mec is closely correlated with methicillin resistance and, therefore, if PSM-mec is detected in a *S. aureus* clinical isolate, then resistance to methicillin can be inferred.

The specificity of predicting mecA carriage in *S. aureus* by detection of the 2415 ± 2 m/z peak is very high and can reach 100% (Rhoads et al., 2016). In 2016, Bruker has released a software that automates this analysis, called “MBT subtyping module,” which is specifically designed to detect the PSM-mec peptide in *S. aureus*, thus obtaining indirect detection of methicillin resistance.

Another application of MALDI-TOF MS has been described for the detection of antimicrobial resistance determinants based on the identification of plasmids bearing *bla_KPC_* carbapenemase genes (Lau et al., 2014), conferring resistance to carbapenems. Plasmid identification by MALDI-TOF MS can be accomplished in a few minutes and, ultimately, provides clinically useful information, given the widespread diffusion of this kind of antibiotic resistance.

A recent method proposed by Idelevich et al. (2017) is based on the possibility to measure the metabolic inhibition, evaluated as decrease in protein synthesis induced after exposure of microorganisms to antimicrobial drugs. The assay is performed directly on the target plate used for MS analysis; it is rapid, yielding results within 5 h, and is not limited to a single class of antibiotics.

In the overall, the discriminatory power of MALDI-TOF MS in strain typing is presently still under investigation (Sanguinetti and Posteraro, 2016), and further studies will be needed to assess whether new methods for detection of antibiotic resistance by MALDI-TOF MS can be effectively applied as routine assays in clinical microbiology laboratories.

## Identification of Fungal Pathogens and Antifungal Susceptibility Testing

The incidence of invasive fungal infections has steadily increased during the last decades (Benedict et al., 2017; Cornely et al., 2017; Pana et al., 2017), mainly due to the growing number of immunocompromised patients and, hence, the prolonged use of broad spectrum antibiotics. Besides the most commonly isolated species, such as *Candida* *albicans* and *Aspergillus* spp., a number of emerging fungal pathogens has been isolated with increased frequency, including *Candida glabrata* (Cleveland et al., 2012; Pfaller et al., 2014), *Trichosporon* spp. (Davies and Thornton, 2014), and filamentous fungi such as *Scedosporium* spp., *Fusarium* spp., and *Mucorales* (Walsh and Gamaletsou, 2013; Davuodi et al., 2015).

Matrix-assisted laser desorption/ionization MS fingerprint represents a robust tool for routine rapid identification of clinically relevant fungi (Marklein et al., 2009; Cassagne et al., 2011; Posteraro et al., 2012; Bader, 2013; Chen et al., 2013; Becker et al., 2014; de Almeida Junior et al., 2014; Triest et al., 2015). However, due to their thick cell wall, obtaining high-quality MALDI-TOF MS spectra of fungal pathogens is more challenging than for most bacterial species and requires optimization and standardization of culture media, growth conditions and extraction procedures. To obtain high quality MALDI-TOF mass spectra, fungal cells may need to be lysed in 70% formic acid and/or by mechanical disruption in a bead-beater (Amiri-Eliasi and Fenselau, 2001; Hettick et al., 2008; Cassagne et al., 2011).

Systemic infections due to *Candida* spp. are characterized by high mortality rates, especially when associated to septic shock and if an effective antifungal therapy is not timely administered (Kollef et al., 2012). Since different *Candida* species are characterized by different susceptibility profiles to antifungal agents, a rapid species identification may have an impact on the patients’ outcomes. Correct identification rates by MALDI-TOF MS directly on positive BC have been correlated with both the protocol used and BC fungal load (Ferreira et al., 2011; Yan et al., 2011; Spanu et al., 2012; Nonnemann et al., 2013).

**FIGURE 1:**
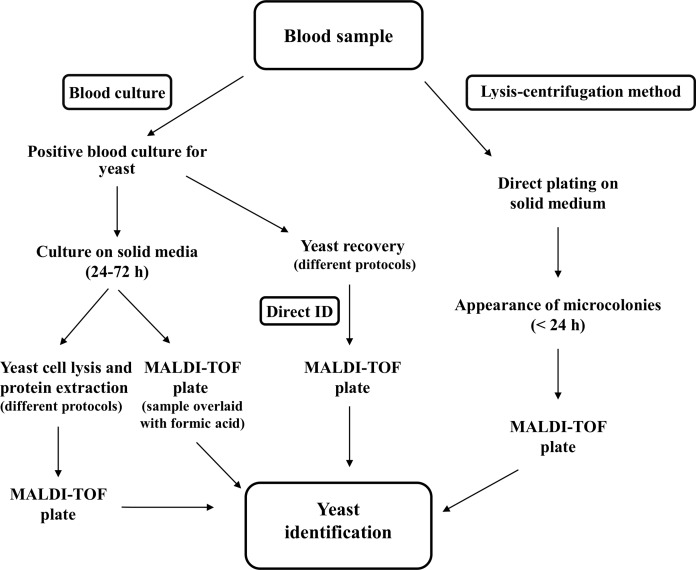
Schematic representation of the different procedures described for identification of yeasts in blood samples by MALDI-TOF MS.

A combined approach was applied to very short-term BCs by evaluating MALDI-TOF MS identification while using the same yeast cell pellets to inoculate Vitek 2 cards for antifungal susceptibility testing (AFST) (Idelevich et al., 2014). AFST results were obtained for 72.7% of positive BCs directly tested by Vitek 2 and essential agreement was 88.6%. However, for some of the isolates the test aborted, and performance for azole susceptibility testing was sub optimal (Idelevich et al., 2014). An alternative method is based on plating small BC aliquots, after lysis, onto solid media before BCs became positive, followed by MALDI-TOF MS analysis of yeast microcolonies (Idelevich et al., 2016). This method allowed identifying different *Candida* spp. in spiked blood samples; however, the applicability of this method in a routine laboratory workflow should be evaluated. A schematic representation of the different methods proposed for yeast identification in blood samples is reported in **Figure 1**.

Another interesting approach for a rapid diagnosis of candidaemia is based on detection of specific biomarkers in sera of patients with invasive candidiasis (Sendid et al., 2015; Mery et al., 2016). In particular, by MALDI-TOF MS analysis, Sendid et al. (2015) identified a disaccharide corresponding to a specific peak of m/z 365 in sera of patients with candidaemia. Further studies will help to assess the clinical utility of this biomarker for an early diagnosis of candidaemia.

A promising method to detect specific resistance to antifungal agents is based on the comparison of MALDI-TOF MS spectra of a yeast isolate after incubation with high, intermediate or null antifungal concentrations (Vella et al., 2013; Saracli et al., 2015). Using this approach, Saracli et al. (2015) evaluated the resistance to triazoles in *C. albicans, C. tropicalis*, and *C. glabrata*: essential agreement between MALDI-TOF MS and conventional AFST ranged between 54 and 97%, and the reproducibility of the MALDI-TOF MS assay between 54.3 and 82.9%, depending on the triazole drug and *Candida* species. However, the time saving using this method over the reference AFST method (Clinical and Laboratory Standards Institute, 2012) was modest (overnight vs. 24 h). In a similar study, a 3-h incubation showed to be sufficient for detection of resistance to caspofungin (CSF) in *C. albicans* isolates (Vella et al., 2013). Further studies will show whether such a short-time incubation may be sufficient to yield accurate and reliable AFST results.

## Concluding Remarks

Matrix-assisted laser desorption/ionization MS has provided a new approach for rapid, reliable, and cost-effective microbial identification. Several studies have shown that the accuracy of MALDI-TOF MS in microbial identification is comparable or superior to automated systems based on biochemical and other phenotypic tests. Indeed, at present many laboratories rely on MALDI-TOF MS for routine identification of microbial pathogens, and can use automated ID systems to confirm uncertain (low-score) identifications, thus achieving correct ID rates very close to 100%, in the overall. Other applications of MALDI-TOF MS in diagnostic microbiology, such as direct sample analysis, prediction of antimicrobial resistance and strain typing, are being developed and evaluated, exploring the possibilities and limits of this technology.

## Author Contributions

WF and AL: conceptualization. WF and AT: manuscript draft. WF, SB, EG, and AL: manuscript revision.

## Conflict of Interest Statement

The authors declare that the research was conducted in the absence of any commercial or financial relationships that could be construed as a potential conflict of interest.

## Footnotes

1 http://www.biomerieux-usa.com/clinical/vitek-ms-healthcare
